# Supplementary material for: Fluid flow drives phenotypic heterogeneity in bacterial growth and adhesion on surfaces
Source: Nat Commun. 2024 Jul 22;15:6161. doi: 10.1038/s41467-024-49997-1 (PMC11263347; doi:10.1038/s41467-024-49997-1)
Supplement: Supplementary file 1 — Supplementary information [file 41467_2024_49997_MOESM1_ESM.pdf]

Supplementary information: Fluid flow drives phenotypic heterogeneity in bacterial growth and adhesion on surfaces

Antoine Hubert[1], Hervé Tabuteau[2], Julien Farasin[1], Aleksandar Loncar[1], Alexis Dufresne[3], Yves Méheust[1] and Tanguy Le Borgne[1]

[1]*Géosciences Rennes, UMR 6118 University of Rennes and CNRS, Rennes, France*
[2]*Institut de Physique de Rennes, UMR 6251 University of Rennes and CNRS, Rennes, France*
[3]*ECOBIO, UMR 6553 University of Rennes and CNRS, Rennes, France*

Supplementary movies

Supplementary movies SupplementaryMovie1.mp4, SupplementaryMovie2.mp4, SupplementaryMovie3.mp4 and SupplementaryMovie4.mp4 show time lapse images of bacteria distributions every 10 minutes for the ulow, low, med and high regimes respectively, with colors corresponding to the time interval of division, using the same color scale as in Fig. 1.

Supplementary data

The dataset used for the main analysis and results of the study is available in the zenodo repository: https://zenodo.org/records/11426128. This includes 3 sets of raw experimental data, each corresponding to independent experiments performed in the ulow, low, med, and high shear stress regime. Each of these data sets is bundled into a single .avi movie, consisting of raw images recorded at a 1/60 Hz acquisition frequency (i.e., 1 image per minute). In addition, source data for the high frame rate statistics (Figure 3) are provided, including detachment ratio, attachment ratio, growth rates and the respective uncertainty estimates for the different regimes.

Supplementary Software

The Matlab and python codes used to analyze raw images and derive bacterial statistics are provided with documentation and examples in the zenodo repository: https://zenodo.org/records/11426128. This includes:

- A Python code based on the Scipy and Trackpy libraries to track particles in time, detect division events, and compute the mean square displacement (MSD) along bacterial trajectories.
- A Matlab code to partition the bacterial populations according to the 40 min time intervals of their birth, compute growth laws for the bacterial population according to their birth time intervals, compute the temporal evolution of MSDs and distinguish dividers from non-dividers at all times.

Supplementary Figures

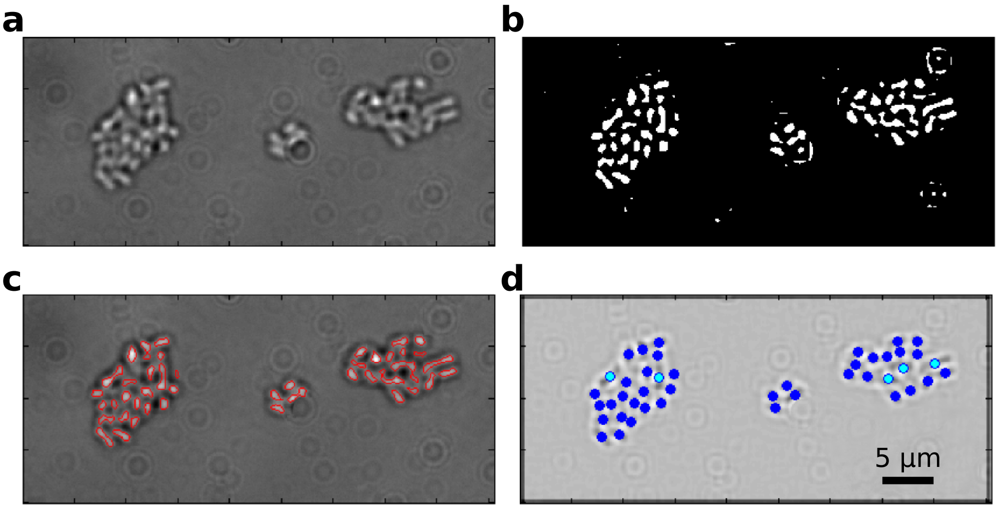

Figure S1: Main steps of image processing. (**a**) raw image, (**b**) binary image after background removal, spatial filtering and intensity segmentation, (**c**) bacterial perimeters found with the binary regions analysis and (**d**) association to all such perimeters of a count of either 1 (dark blue) or 2 (light blue) individual bacteria depending on region area. Grey scales on images (a) and (c) are raw data in pixel intensity on a 16 bit depth scale (0 to 65565), image (b) is binary (0 or 1) and grey scale on image (d) are in pixel intensity after applying the background subtraction and spatial filter.

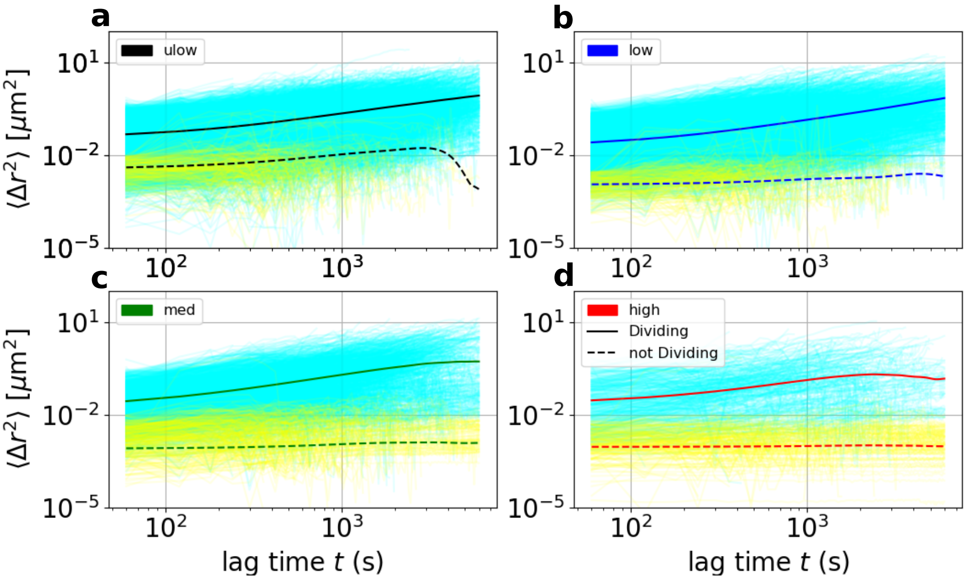

Figure S2: Segregation of dividers and non-dividers. Mean Squared Displacement (MSD, $\langle \Delta r^2 \rangle$) of the centroids of ellipsoids fitted around bacteria for the different flow regimes (table 1): **a** ulow, **b** low, **c** med and **d** high. Blue curves correspond to dividers, with a final MSD higher than 0.25 µm^2. Yellow curves correspond to the other category, the non-dividers. Plain lines (−) correspond to the mean MSD of dividers and dashed lines (−−) to the mean MSD of non-dividers.

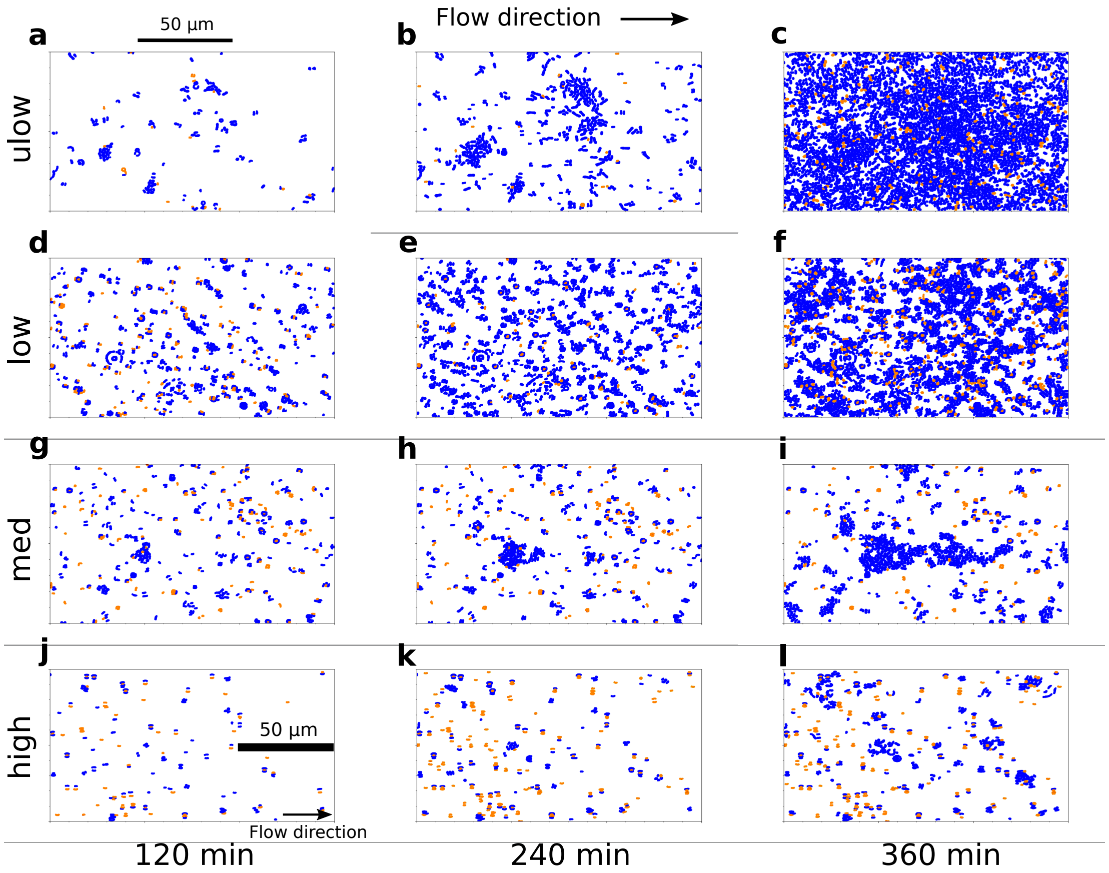

Figure S3: Snapshots of dividers and non-dividers for the four different regimes: ulow (**a-c**), low (**d-f**), med (**g-i**) and high (**j-l**), at times 120 (left row), 240 (middle row) and 360 (right row) minutes after the start of the experiments. Dividers are colored in blue, non-dividers in orange. Dividers produce non-dividers stochastically, leading to an increase in the number of non-dividers with time (Fig. 4b).

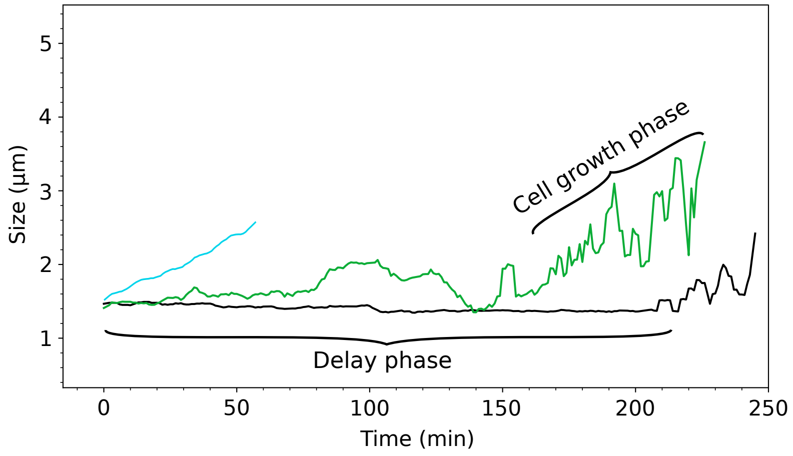

Figure S4: Lagged dividers dynamics. Example of bacteria elongations for lagged dividers (purple, green and black lines) compared to a continuous divider (blue line). Lagged dividers characterized by a delay phase with constant size followed by an elongation phase similar to that of continuous dividers.

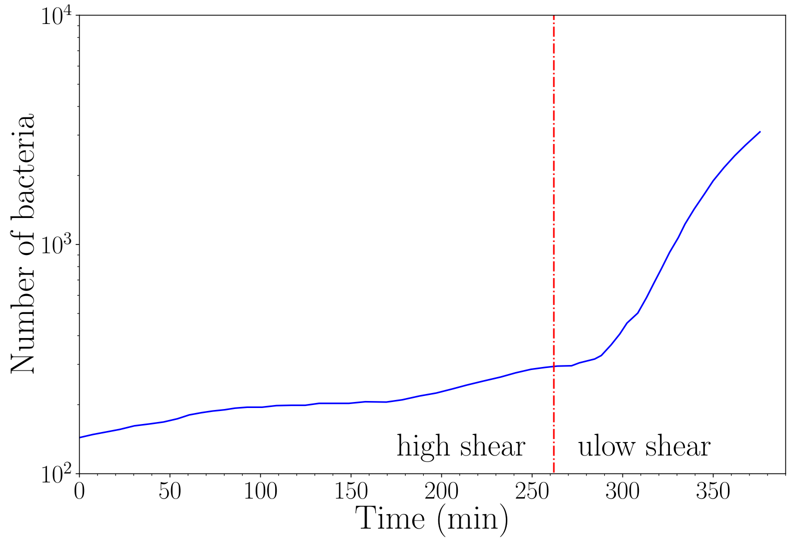

Figure S5: Evolution of the number of attached bacteria when imposing a sudden change in the imposed shear, from high to ulow. Number of bacteria attached to the surface as a function of time. The flow rate is changed from high shear to ultra low shear after 260 minutes (red line).
