# Supplementary material for: Fluid flow drives phenotypic heterogeneity in bacterial growth and adhesion on surfaces
Source: Nat Commun. 2024 Jul 22;15:6161. doi: 10.1038/s41467-024-49997-1 (PMC11263347; doi:10.1038/s41467-024-49997-1)
Supplement: Supplementary file 3 — Description of Additional Supplementary Files [file 41467_2024_49997_MOESM3_ESM.pdf]

Description of Additional Supplementary Files:

Supplementary Movie 1: Time lapse images of bacteria distributions every 10 minutes for the ulow regime, with colors corresponding to the time interval of division, using the same color scale as in Fig. 1.

Supplementary Movie 2: Time lapse images of bacteria distributions every 10 minutes for the low regime, with colors corresponding to the time interval of division, using the same color scale as in Fig. 1.

Supplementary Movie 3: Time lapse images of bacteria distributions every 10 minutes for the med regime, with colors corresponding to the time interval of division, using the same color scale as in Fig. 1.

Supplementary Movie 4: Time lapse images of bacteria distributions every 10 minutes for the high regime, with colors corresponding to the time interval of division, using the same color scale as in Fig. 1.
